# Lifestyle Factors Involved in the Pathogenesis of Alopecia Areata

**DOI:** 10.3390/ijms23031038

**Published:** 2022-01-18

**Authors:** Yoko Minokawa, Yu Sawada, Motonobu Nakamura

**Affiliations:** Department of Dermatology, University of Occupational and Environmental Health, Kitakyushu City 807-8555, Japan; mino-yoko@med.uoeh-u.ac.jp (Y.M.); motonaka@med.uoeh-u.ac.jp (M.N.)

**Keywords:** alopecia areata, lifestyle factors, Th1, Th2, Th17

## Abstract

Alopecia areata is a representative inflammatory skin disease that is associated with various environmental stimuli. While psychological stress is believed to be a major pathogenetic trigger in alopecia areata, infants and newborns also suffer from the disease, suggesting the possible presence of other environmental factors. Daily lifestyle is well known to be involved in various inflammatory diseases and influences the severity of inflammatory skin diseases. However, only a limited number of studies have summarized these influences on alopecia areata. In this review article, we summarize lifestyle factor-related influences on the pathogenesis of alopecia areata and focus on environmental factors, such as smoking, alcohol consumption, sleep, obesity, fatty acids, and gluten consumption.

## 1. Introduction

The skin is the outermost layer of the body that is exposed to various environments [1,2]. Because of this characteristic, the skin plays various vital roles, such as protection against external stimuli and the exertion of inflammatory cytokines [3,4]. Recent studies have identified that daily lifestyle factors, such as smoking, alcohol intake, and sleep, play a vital role in the development of inflammatory skin diseases [5].

The hair follicle is a representative component of the scalp that is strongly influenced by hormones and immune cells [6,7]. Hair follicles are mainly located at the head of the scalp, which is expected to be affected by various factors, such as ultraviolet light exposure and temperature. Alopecia areata is a representative hair follicle disease, and environmental factors are known to influence disease development [8]. However, articles regarding detailed information focused on daily lifestyle factors that influence the pathogenesis of alopecia areata are limited.

Because of the immunological pathogenesis of alopecia areata, various therapeutic options are currently available to regulate hair follicle inflammation. Consistently, topical steroid and immunomodulator therapy with squaric acid dibutylester (SADBE) are effective treatments [9]. Environmental factors influence immunological actions in various inflammatory diseases; therefore, daily lifestyle factors are also associated with the development of alopecia areata.

In this review, we summarize the influence of daily lifestyle-related environmental factors on the development of alopecia areata. This review introduces epidemiological data and mechanistic findings of lifestyle factor-related alopecia areata. Furthermore, we discuss the possible pathogenetic role of these environmental factors in alopecia areata based on the immunological pathology of the disease.

## 2. Alopecia Areata

Alopecia areata is a common form of immune-mediated alopecia in which the autoimmune attack of the hair follicle results in non-scarring hair loss, which is characterized by a range of circular patches on the scalp. The estimated lifetime risk of alopecia areata has been reported to be 1.7–2.1% [9]. Approximately 20% of cases are children, with 60% of alopecia areata patients recognizing their first hair loss patch before 30 years of age [10]. A higher prevalence of alopecia areata has been reported in patients aged 10–25 years (60%) [11,12]. The exact cause of alopecia areata remains unknown. Current studies provide evidence supporting an autoimmune response to hair follicles, in addition to unknown environmental influences. Psychological stress has been proposed as an environmental factor that contributes to the development of alopecia areata. A previous study reported that at least 23% of patients experienced an emotional event or crisis before the onset of alopecia areata [13]. In contrast, psychological stress alone cannot explain the complete pathogenesis of alopecia areata because newborns and infants sometimes experience the disease [14]. Therefore, other factors, such as infections, toxins, and even food, are thought to be associated with autoimmune dysregulation processes, which have been proposed as possible triggers of the disease, although not all cases have been validated.

## 3. The Immune Escape Mechanism in the Normal Hair Follicle

**Figure 1:**
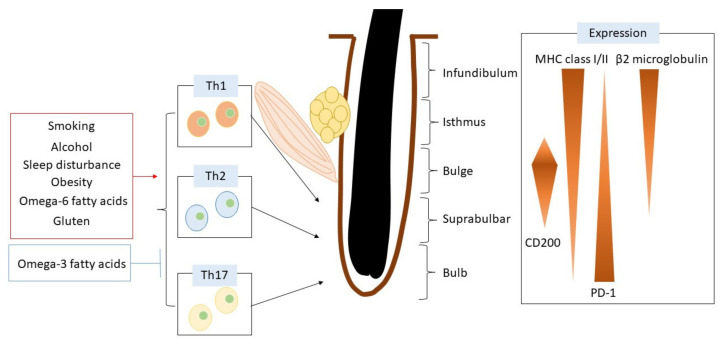
Hair follicle structure and regulatory mechanisms of immune reaction related to lifestyle factors.

The hair follicle is an immunologically privileged part of the skin that can escape from an immune cell attack (Figure 1). The reduction of MHC class I and β2 microglobulin expression in the hair bulb contributes to the suppression of immune cell activation in hair follicles [15]. Hair follicles bearing CD200 also downregulate the function of antigen-presenting cells. CD200 is decreased in bulge sites in patients with alopecia areata [15]. Programmed death ligand-1 (PD-L1) is upregulated in dermal sheath cup cells [16]. PD-L1 is essential for the immune escape phenomenon, and anti-PD-L1 antibody treatment triggers alopecia areata [17].

## 4. The General Inflammatory Action in Hair Follicles of Alopecia Areata Patients

Histological examination revealed inflammatory cell infiltrates around the bulbar region of hair follicles in patients with alopecia areata [18]. Hair shaft cortical differentiation is an essential process that the hair matrix epithelium undergoes. The early stage of this differentiation process is a fragile term for the development of hair growth; therefore, hair growth is affected by immune cell reactions during this period, leading to vacuolar degeneration in anagen follicles and impairment of hair shaft strength. MHC class I and class II expression in the pre-cortical hair shaft is enhanced following immune cell reactions to hair follicles [19,20].

Hair follicles are considered as structures with an ability to escape from the autoimmune reaction by enhancing the suppressive signaling around them to impair CD8+ cell and NK cell function. Therefore, it is speculated that the braking of this suppressive action is a major cause of alopecia areata. Lower expression of MHC class I and class II, as well as macrophage migration inhibitory factor (MIF), are thought to inhibit T cell and CD56+ NKG2D+ NK cell function [20,21]. In contrast, CD56+ NKG2D+ NK cells infiltrate around the hair follicles in patients with alopecia areata [22].

The presence of autoantigens has also been investigated in alopecia areata. The epitopes derived from a hair shaft structure protein, trichohyalin, and pigmentation-associated tyrosine-related protein 2 enhance CD8+ cell infiltration in patients with alopecia areata [23,24,25], suggesting possible involvement of keratinocyte-derived antigens and/or melanin-related proteins.

## 5. Other Immune Cell Orchestrations in the Pathogenesis of Alopecia Areata

Recent studies have also identified the roles of other inflammatory cytokines in alopecia areata [26]. In addition to Th1, cytokines such as IFN-gamma, Th2 cytokines, and especially IL-13 are also upregulated in alopecia areata [26]. IL-23 is enhanced in alopecia areata [26]. In addition, CD4+ IL-17+ cells infiltrate around hair follicles in the acute phase [27] and are significantly higher in patients less than 30 years old [28], while Th17 is downregulated in the late phase [29]. These findings indicate that Th1, Th2, and Th17 activation is involved in the development of alopecia areata.

## 6. Daily Lifestyle Factors Related to Alopecia Areata

### 6.1. Smoking

Tobacco is a plant native to the tropics of the genus *Nicotiana tabacum*. As a leaf component, it contains nicotine, which has a strong addictive effect on human health. In addition, various chemicals are contained in tobacco, and tobacco-smoke exposure causes various harmful effects on human health. Consistently, smoking influences various inflammatory skin diseases [5] and affects the development of alopecia.

Several epidemiological studies have investigated the risk of alopecia following smoking. Current smokers showed a higher risk of alopecia areata incidence than non-smokers, with a hazard ratio of 1.88 [30]. The duration and volume of tobacco smoking were also related to the risk of alopecia areata. A greater than 10-year smoking history showed an increased hazard ratio (2.25) for alopecia areata, and a smoking volume of more than five cigarettes per day also showed a significantly higher hazard ratio (2.03) for alopecia areata [30]. 

Although the detailed smoking-related pathogenesis of alopecia remains unclear, cigarette smoke increases the production of various inflammatory cytokines and decreases the levels of anti-inflammatory cytokines. 

Smoking activates Th17-mediated skin inflammation and increases IL-17-producing cell frequency in the peripheral blood and organs [31]. An imbalance in Th17/Treg differentiation may adversely affect the homeostasis of the hair follicle infundibulum [32]. Therefore, smoking may also activate IL-17-producing cells in the skin. Therefore, Th17-mediated inflammation in hair follicles might be involved in the pathogenesis of alopecia areata.

Smoking exacerbates the Th2 immune response in the skin and enhances inflammatory skin reactions in atopic dermatitis [33]. Smoking increases the levels of Th2 inflammatory cytokine IL-13 [34] and enhances Th2 polarization in an ERK-dependent mechanism [35], suggesting that the Th2-mediated immune response in alopecia areata might be exacerbated by smoking exposure. 

The Th1 cutaneous immune response is also enhanced by smoking [36]. Smoking activates inflammatory cytokine production and IFN-γ and exacerbates inflammatory responses. 

In total, inflammatory cytokines were exacerbated after smoking exposure. Therefore, it is necessary to avoid direct smoking in patients with alopecia areata. Free radicals are involved in the development and worsening of alopecia areata. Cigarette smoke contains a high concentration of free radicals, which may build up in the hair follicle, eventually leading to a breakdown of immune privilege [37,38]. In addition, indirect smoking exposure is also involved in the development of skin inflammation. Smoking exposure during childhood increases the risk for atopic dermatitis [39,40], suggesting that family cooperation is also important to avoid indirect smoking exposure in houses.

### 6.2. Alcohol Consumption

A statistical analysis was conducted to elucidate the possible role of alcohol in the pathogenesis of alopecia areata. Regular drinkers showed a lower risk of alopecia areata with a hazard ratio of 0.49 [30]. However, this result might need to reflect alcohol-mediated skin inflammation because several studies have shown that alcohol exacerbates skin inflammation in patients with atopic dermatitis [41] and psoriasis [42].

Ethanol-exposed psoriatic skin taken from patients enhances the production of IFN-γ, TGF-α, and IL-6 secretion by the lymphocyte cell line [43]. In addition, lymphocytes from psoriasis patients enhance proliferation by alcohol exposure, which cannot be observed in healthy subjects [44]. Therefore, these findings suggest that Th17-dominant cutaneous inflammation can be exacerbated by alcohol exposure, and that Th17-mediated alopecia areata might be directly influenced by alcohol exposure.

A recent statistical analysis showed a close relationship between alcohol consumption and the risk for atopic dermatitis. Alcohol consumption during pregnancy increases the risk of pediatric atopic dermatitis [41]. The alcohol metabolite acetaldehyde exposure enhances histamine release by mast cells [45] and causes itching and skin inflammation [46].

These findings suggest that alcohol consumption may be associated with the immunological risk of alopecia areata. In contrast, psychological stress is closely related to the development of alopecia areata [47,48] while alcohol consumption relieves psychological stress [49], which might negatively regulate the development of alopecia areata. Mild intoxication with ethanol impairs the ACTH and cortisol secretion response to intravenous CRH administration [50], suggesting that alcohol consumption impairs the pathogenesis of alopecia areata. 

In contrast, a high dose of ethanol did not alter the levels of these stress hormones, indicating that an appropriate volume of alcohol intake might be better for alopecia areata. However, the long-term efficacy of alcohol should be determined in further investigations. 

### 6.3. Sleep Disturbance

One study investigated the risk of developing alopecia areata in patients with sleep disorders [51]. A total of 25,800 patients with sleep disorders and 129,000 control subjects were enrolled in this study. Patients with sleep disorders showed an increased risk of alopecia areata with a hazard ratio of 1.651, and this tendency was especially observed in younger age groups, under 45 years of age. Multivariate analysis also showed that sleep disorders are associated with the risk of developing alopecia areata with an odds ratio of 1.913, in addition to other autoimmune diseases, such as rheumatoid arthritis and Hashimoto thyroiditis.

Another study also investigated the risk of alopecia in patients with sleep disorders [52]. A total of 5648 patients with alopecia areata and 22,592 matched controls were enrolled in this study. Sleep disorders were associated with an increased risk of alopecia areata, with a hazard ratio of 4.70. Both obstructive sleep apnea and non-apnea insomnia also increased the risk of alopecia areata with hazard ratios of 3.89 and 4.77, respectively.

On the contrary, one study showed that sleep quality seems to have no relationship with the risk of alopecia areata [53]. This study evaluated sleep quality using a self-administered questionnaire, the Epworth Sleepiness Scale, to evaluate excessive daytime sleepiness. Approximately 11.4% of alopecia areata patients suffered from excessive daytime sleepiness. The mean Epworth Sleepiness Scale score showed no significant difference between alopecia areata and healthy subjects and no correlation with the severity and duration of alopecia areata. Therefore, further large-scale studies are necessary to determine the actual impact of sleep disturbance on the risk of alopecia areata.

Several studies have investigated the mechanisms of sleep disturbance in alopecia areata. Contact hypersensitivity reaction was measured in mice with CLOCK gene mutations to evaluate the influence of biological clock dysfunction, which showed enhanced ear swelling response and mast cell infiltration in the skin [54], indicating that sleep disturbance might lead to a Th1 immune response in alopecia areata.

Sleep disturbances influence the skin’s physiological functions and cause dryness and itching [55]. The quality of sleep disturbance is correlated with the severity of atopic dermatitis [56]. The human circadian rhythm is regulated by clock genes, with typical examples being CLOCK and BMAL1. Aquaporin 3 acts as a water pump in the epidermis and enhances skin moisturization. Aquaporin 3 expression is regulated by the circadian clock gene, and disruption of the circadian rhythm impairs the function of aquaporin 3, leading to dysfunction in epidermal moisturization [56]. There is evidence that circadian clock genes are highly expressed in hair germ progenitors in early anagen. For instance, Clock or Bmal1 deficiency delays anagen progression through the prevention of germ progenitor cell cycle progression in the G1 phase [57]. Moreover, this finding might be related to the aggravation of alopecia areata due to sleep disturbances.

Shift work increases the risk of psoriasis [58], suggesting that sleep disturbance influences the development of the Th17-mediated skin immune response. Deficiency of clock genes causes biological clock dysfunction and enhances the imiquimod-induced cutaneous Th17 immune response due to impairment of IL-23 receptor expression [59]. Sleep disturbances influence various immunological reactions in the skin and may influence the development of Th17-mediated alopecia areata. 

### 6.4. Obesity

Subcutaneous fat is essential for storing energy to survive under starvation conditions. However, excess total energy intake increases energy storage in subcutaneous fat and causes obesity. Obesity exacerbates various harmful human diseases, especially inflammatory diseases [60]. Furthermore, obesity increases the risk of alopecia areata (OR: 1.15) [61]. However, the detailed mechanism of obesity-related alopecia areata remains unclear. 

Previous clinical data and experimental models have demonstrated the involvement of adipokines in the pathogenesis of various autoimmune diseases, suggesting that obesity may be a major environmental factor contributing to the onset and progression of autoimmune diseases, including alopecia areata [62]. In addition, there are a couple of recent studies showing a possible link between alopecia areata and adipokines, especially adiponectin and leptin [63,64].

The Th17 immune response depends on obesity. A high body mass index is positively associated with psoriasis risk [65]. Mice fed with a high-fat diet showed increased IL-17-mediated skin inflammation by imiquimod treatment and enhancement of the production of IL-17 producing cells even in the steady state [60], suggesting that the IL-17-dominant baseline condition is involved in the mechanism of obesity-related Th17 cutaneous inflammation.

Obesity is also associated with Th2-mediated skin diseases, especially atopic dermatitis [66]. A higher body mass index was positively correlated with the risk of atopic dermatitis (OR: 1.02). This study also indicated an increased risk of atopic dermatitis by approximately 2% for each 1 kg/m^2^ ascending body mass index. Consistently, a high-fat diet in obese mice increased TSLP production and enhanced the Th2 immune response in a murine model of eosinophilic esophagitis [67], which is also expected in atopic dermatitis. Furthermore, skin barrier function is impaired in obesity [68]. These findings suggest that obesity is also responsible for the development of a cutaneous Th2-mediated immune response possibly related to the pathogenesis of alopecia areata.

The cutaneous Th1 immune response is enhanced in obese individuals. Obesity causes lymphatic vessel dysfunction and enhances lymphatic fluid leakage from capillary lymphatic vessels [69]. This alteration increases the Th1-mediated contact hypersensitivity response and delays clearance of skin inflammation [70]. Therefore, the Th1-mediated development of alopecia areata might be enhanced by obesity.

### 6.5. Fatty Acids

Fatty acids are a component of the major structure of the cell membrane and regulate the signaling pathway in cell interaction [71]. Recent studies have updated the importance of fatty acids in the pathogenesis of various diseases and have determined their possible therapeutic efficacy. Among the various fatty acids, omega-3 and omega-6 fatty acids are recognized as representative fatty acids in the human body. Omega-3 fatty acids, such as eicosatetraenoic acid (EPA) and docosahexaenoic acid (DHA), are abundant in fish oil. Epidemiological studies have shown that omega-3 fatty acids reduce the risk of inflammatory diseases [72]. Increased intake of omega-3 fatty acids and fish oil is negatively correlated with the incidence of Th2 allergic diseases [73]. In contrast, omega-6 fatty acids are metabolized to arachidonic acid and subsequently converted into inflammatory lipid mediators, such as leukotriene B4 (LTB4) and prostaglandin E2 (PGE2), which contribute to the development of inflammatory skin diseases.

The first report of possible involvement of fatty acids in alopecia areata and alopecia-like hair loss was observed in COX-2 overexpressed in K14 mice [74]. The expression of COX-2 was decreased in catagen, repressed in telogen, and was reactivated in the basal outer root sheath and basal sebaceous gland cells of anagen hair follicles [75]. COX-2 overexpression in keratin-5 mice slowed the catagen phase of the hair follicle and subsequently disturbed the hair follicle cycle, leading to alopecia, which was improved by oral administration of the COX-2 inhibitor valdecoxib [75]. Prostaglandin D2 (PGD2) is produced in the bald scalp. During the hair follicle cycle, prostaglandin D synthase and PGD2 increase during the regression phase, suggesting their inhibitory effect on hair growth [76]. K14-specific Ptgs2 overexpression mice showed elevated PGD2 levels in the skin and caused alopecia with impairment of hair follicles [76]. Therefore, PGD2 exerts a direct regulatory action on the hair cycle.

The 15-lipoxygenase (ALOX15) is responsible for the generation of specialized pro-resolving lipid mediators that play essential roles in anti-inflammatory actions. Alox15-deficient mice show hair loss and increased inflammatory cell infiltration around the hair follicles, which are impaired by resolvin D2 treatment [77].

These findings suggest that daily intake of omega-3 fatty acids might have a beneficial effect in alopecia areata; however, only one report identified a statistically significant efficacy of daily intake of omega-3 PUFAs in patients with alopecia areata. 

Since there are a limited number of studies focusing on the direct role of fatty acids in the pathogenesis of alopecia areata, we discussed the possible role of fatty acids in immune regulation of Th1, Th2, and Th17-mediated cutaneous immune responses, which are involved in the development of alopecia areata. DHA and EPA administration reduces skin inflammation, production of inflammatory cytokines, and inflammatory cell infiltration in Th1-mediated contact hypersensitivity response [78]. An EPA metabolite, resolvin E1, impairs dendritic cell migration in the skin and subsequently suppresses Th1-mediated immune response and skin inflammation [79]. In contrast, the importance of omega-6 fatty acids has also been reported. PGE2 enhances the pathogenesis of contact hypersensitivity through specific receptors. The PGE2-EP3 signaling pathway exacerbates inflammatory skin reactions in contact hypersensitivity [80], and PGE2-EP4 signaling also enhances inflammatory skin reactions mediated by activation of Langerhans cell migration and activation [81].

LTB4 is abundantly observed in psoriatic lesional skin, and LTB4 receptor BLT1-deficient mice impair imiquimod-induced Th17 skin inflammation by suppression of IL-17, producing cell migration and production of IL-23 by dendritic cells [82]. BLT1-deficient mice also show impaired neutrophil migration [83]. Thromboxane A2 enhances IL-17 production and enhances imiquimod-induced cutaneous IL-17 immune reactions [84].

EPA suppresses the production of inflammatory cytokines and the inflammatory lipid mediators LTB4 and PGE2 [85]. Because a lower intake of omega-3 fatty acids was observed in psoriasis patients [86], the dietary intake of omega-3 fatty acids is expected to suppress the Th17-mediated immune response. Indeed, omega-3 fatty acid metabolites showed anti-inflammatory actions in imiquimod-induced IL-17-mediated skin inflammation [82,87,88].

DNA/EPA administration impaired LTB4 production and Th2 skin inflammation [89], and the EPA metabolite resolvin E1 suppressed atopic dermatitis-like skin inflammation [90].

Atopic dermatitis skin contains a high amount of LTB4 [91]. High amounts of omega-6 fatty acid intake during prenatal infants increases the relative risk of atopic dermatitis in children (OR: 1.25) [92]. LTB4 enhances the migration of neutrophils and Th2 cells in the skin [93], indicating the pathogenic role of LTB in the cutaneous Th2 immune response. 

A high amount of PGE2 is also observed in skin with atopic dermatitis [91], suggesting a role of PGE2 in the pathogenesis of atopic dermatitis. Previous studies have provided us with a possible beneficial effect of PGE2 in atopic dermatitis. COX-2 inhibitors exacerbate Th2-mediated immune responses in the skin [94]. A recent study showed that the PGE2-EP2 signaling pathway downregulates PAR2 receptor expression in keratinocytes and suppresses TSLP production, which leads to the impairment of Th2-mediated immune response [95], suggesting that PGE2 plays a protective role in Th2-mediated skin inflammation.

### 6.6. Gluten

Gluten is a protein derived from cereal grains and seeds and is recognized as a trigger of celiac disease, which enhances allergic immune responses [96]. Furthermore, gluten exacerbates inflammatory skin diseases in non-celiac disease patients [97], indicating the pathogenic role of gluten in inflammatory diseases. Patients with celiac disease are sometimes complicated with alopecia areata [98] and 70.9% showed an improvement in a group of alopecia areata with celiac disease patients given a gluten-free diet [99]. However, the detailed molecular mechanism behind the phenomenon remains unclear.

A previous study showed an association of gluten antigens with the hair follicle peptide peroxiredoxin 5 (PRDX5) [100], which is one of the genes associated with alopecia areata [101].

Patients with celiac disease show increased IFN-γ production [102], which is impaired by a gluten-free diet [103], suggesting a possible role of gluten in exacerbating Th1-mediated inflammation. 

The prevalence of atopic dermatitis is high in celiac disease [104]. Gluten intake itself increases the risk of atopic dermatitis [105], and gluten enhances cutaneous Th2 immune responses in a mouse experiment [106]. As a mechanism, gluten activates TSLP production by keratinocytes, which promotes the cutaneous Th2 immune response [107], indicating that gluten might worsen alopecia areata by enhancing the Th2 immune response.

Gluten enhances Th17-mediated skin inflammation. Patients with celiac disease concomitantly diagnosed with psoriasis showed improvement of skin eruptions following a gluten-free diet [108]. IL-17 was upregulated in mucosal lesions in celiac disease [109], suggesting that gluten might enhance the IL-17-dominant immune reaction in the skin. These findings suggest that gluten can exacerbate hair follicle inflammation involved in the immunological pathogenesis of alopecia areata.

## 7. Conclusions

**Figure 2:**
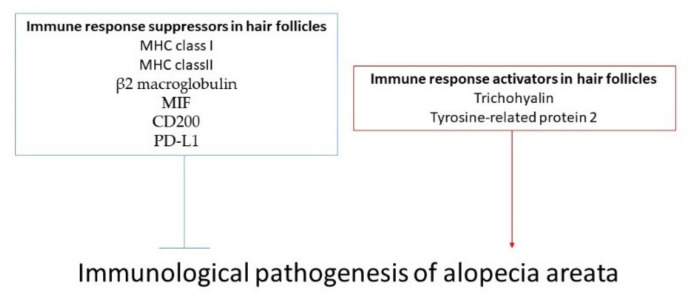
Immunological regulators in the hair follicle associated with alopecia areata. There are immune response suppressors in hair follicles, such as MHC class I/II, β2 macroglobulin, MIF, CD200, and PD-L1. These immunosuppressive factors are downregulated in hair follicles of patients with alopecia areata. On the other hand, trichohyalin and tyrosine-related protein 2 are known as immune response activators in hair follicles and are also related to the pathogenesis of alopecia areata.

We showed that various daily lifestyle factors are involved in the pathogenesis of alopecia areata (Figure 2). The investigation of daily lifestyle factors may be helpful to obtain a better understanding of the pathogenesis of alopecia areata in an individual patient. Because alopecia areata drives various immunological conditions during the clinical course, the detailed molecular mechanism involved in the daily lifestyle-related pathogenesis of alopecia areata remains unclear. In addition, the effort needed to maintain a healthy lifestyle in patients with alopecia areata might cause psychological distress and lead to nervousness, which is one of the major triggers for alopecia areata. Because there is limited clinical evidence regarding daily lifestyle factors affecting alopecia areata, we could not recommend excess guidance of daily lifestyle in patients with alopecia areata. Therefore, further clinical trials or epidemiological studies based on the findings obtained from in vivo or in vitro experiments are needed to elucidate molecular mechanisms of the disease and might be helpful in guiding how to institute appropriate lifestyle guidance in patients with alopecia areata in the future. 

The hair follicle is divided into five sites: infundibulum, isthmus, bulge, suprabulb, and bulb sites. To protect against autoimmune reactions, hair follicles differentially express various immunomodulatory molecules. In the pathogenesis of alopecia areata, there is a complex immunological pathology mediated by Th1, Th2, and Th17 cells, which are regulated by various daily lifestyle factors. 

## Data Availability

Not applicable.
